# The human hippocampus beyond the cognitive map: evidence from a densely amnesic patient

**DOI:** 10.3389/fnhum.2014.00711

**Published:** 2014-09-15

**Authors:** Pamela A. Banta Lavenex, Françoise Colombo, Farfalla Ribordy Lambert, Pierre Lavenex

**Affiliations:** ^1^Laboratory for Experimental Research on Behavior, Institute of Psychology, University of LausanneLausanne, Switzerland; ^2^Unit of Neuropsychology and Aphasiology, Cantonal Hospital of FribourgFribourg, Switzerland; ^3^Laboratory of Brain and Cognitive Development, Department of Medicine and Fribourg Center for Cognition, University of FribourgFribourg, Switzerland

**Keywords:** medial temporal lobe, parahippocampal, declarative memory, spatial memory, interference, amnesia, memory capacity

## Abstract

We tested a densely amnesic patient (P9), with bilateral hippocampal damage resulting from an autoimmune disorder, and 12 age- and sex-matched controls on a series of memory tasks designed to characterize allocentric spatial learning and memory abilities. We compared P9's ability to perform spatial memory tasks with her ability to perform non-spatial, color memory tasks. First, P9's performance was impaired as compared to controls even in the simplest versions of an allocentric spatial memory task, in which she had to find repeatedly over 10 trials the same location(s) of one, two or three illuminating foot pad(s) among 23 pads distributed in an open-field arena. In contrast, she performed as well as controls when she had to find repeatedly over 10 trials the same one, two or three pad(s) marked by color cue(s), whose locations varied between trials. Second, P9's performance was severely impaired in working memory tasks, when she had to learn on a trial-unique basis and remember the location(s) or the color(s) of one, two or three pad(s), while performing an interfering task during the 1-min interval separating encoding and retrieval. Without interference during the retention interval of the trial-unique tasks, P9's performance was partially preserved in the color tasks, whereas it remained severely impaired in the allocentric spatial tasks. Detailed behavioral analyses indicate that P9's memory representations are more limited than those of controls both in their precision (metric coding) and in the number of items that can be maintained in memory (capacity). These findings are consistent with the theory that the hippocampus contributes to the integration or binding of multiple items, in order to produce high-resolution/high-capacity representations of spatial and non-spatial information in the service of short-term/working and long-term memory.

## Introduction

### The rodent hippocampus and space

Since the publication of *The Hippocampus as a Cognitive Map* (O'Keefe and Nadel, 1978), countless studies have provided evidence for the role of the rodent hippocampus in allocentric spatial memory (Morris et al., 1982; Morris, 2007). Such an emphasis on the spatial function of the hippocampus is not surprising, given that the initial description of the so-called *place cells* emphasized the role of the rat hippocampus as a spatial map (O'Keefe and Dostrovsky, 1971). Indeed, O'Keefe and Dostrovsky concentrated their description on the response properties of 8 of the 76 units recorded in their experiment. Place cell activity was most strongly influenced by several multimodal cues, which likely allowed the animal to orient in its environment. For one place cell, visual information was found critical to maintain its selective firing, as turning off the light abolished its response. In contrast, other place cells remained active in the dark, attesting to the role that other sensory or memory inputs might play in keeping track of an individual's position in absence of visual cues. However, half of these place cells (4 out of 8) did not fire unless the animal was in a moderate state of arousal, was situated in the correct part of the testing platform and, in addition, was receiving an appropriate sensory stimulus. An example of that stimulus was described as a tactile stimulus produced when the rat was firmly restrained by a hand placed over its back with thumb and index finger on its shoulder and upper arm. Accordingly, the activity of individual hippocampal neurons suggests that the function of the hippocampus extends beyond the elaboration of spatial maps of the environment, to the integration or binding of multimodal sensory information experienced by the animal. Thus, although it can be concluded that in rodents the elaboration of an allocentric, spatial relational representation of the environment requires the hippocampus, it is clear that the rodent hippocampus is not limited to this function (Morris, 2007).

### The hippocampus outside of space

A large number of studies have thus also focused on the role of the hippocampus outside the spatial domain. Olton and colleagues proposed that the hippocampus is selectively involved in behaviors that require *working memory*, a short-term memory that requires *flexible* stimulus-response associations and is highly susceptible to interference, irrespective of the type of material (Olton et al., 1979). Cohen and Eichenbaum advocated that the hippocampal formation is essential for the processing of *relational* information, i.e., representing the arbitrary or accidental relations among the constituent elements of events or scenes (Cohen and Eichenbaum, 1993; Eichenbaum, 2001). Interestingly, as these theories have evolved, they have ultimately supported the early view proposed by Scoville and Milner (1957) that the hippocampus is critical for the memory of current experience or episodic memories (reviewed by Morris, 2007), which are defined as the memories for personal events or episodes that happen in unique spatiotemporal contexts, that is in particular places at particular times (Tulving, 2002). In addition, in their description of patients with severe memory deficits, Scoville and Milner also suggested that the hippocampus is necessary to maintain information over short delays in the presence of distractive information (Scoville and Milner, 1957). Finally, a number of other influential theories of hippocampal function have been proposed that we are unfortunately unable to mention here. These other theories are, however, less relevant to a discussion concerning the role of the human hippocampus in spatial coding, as was tested in the current study and is the subject of this special issue of *Frontiers in Human Neuroscience*.

### Different representations of space

As compared to the well-established role that the rodent hippocampus plays in allocentric spatial memory, clear evidence for the role of the monkey hippocampus in spatial memory is more recent (Banta Lavenex et al., 2006). The previous lack of consensual evidence in non-human primates derived largely from the use of various experimental designs and a lack of consideration of the representational demands of the tasks utilized in those studies, specifically the lack of distinction between egocentric (body-centered; hippocampus-independent) and allocentric (world-centered; hippocampus-dependent) frames of reference (see Banta Lavenex and Lavenex, 2009 for review). In order to avoid such confusion, it is essential to specify the theoretical concepts used to describe “space,” the details of the methodologies used to obtain experimental findings, and the representational demands of different behavioral tasks (Eichenbaum et al., 1990). Here, we use the term *allocentric reference frame* to refer to a psychological framework for representing the *relationships* between multiple objects in the environment (in contrast to simple associations between an object or view/scene and a location, or reference frames defined in relation to the observer (O'Keefe and Nadel, 1978), which can be considered equivalent to a *viewpoint-independent* representation of space (Nadel and Hardt, 2004; Burgess, 2006), a *spatial relational* representation of the environment (Eichenbaum et al., 1990; Banta Lavenex et al., 2006), or as originally stated a *cognitive map* (Tolman, 1948).

### The human hippocampus and space

Similar to the work in rodents and monkeys, a number of neuropsychologists have investigated the role of the human hippocampus in spatial learning and memory in patients with selective brain damage (Morris et al., 1996; Abrahams et al., 1997; Bohbot et al., 1998; Holdstock et al., 2000; Astur et al., 2002; Morris and Mayes, 2004; Parslow et al., 2004, 2005; Maguire et al., 2006; Bohbot and Corkin, 2007; Bartsch et al., 2010; Goodrich-Hunsaker and Hopkins, 2010; Goodrich-Hunsaker et al., 2010). Most human studies have yielded results consistent with those in rodents and monkeys, suggesting that there are multiple forms of spatial knowledge and that the human hippocampus is particularly involved with allocentric (viewpoint-independent), but not egocentric (viewpoint-dependent), representations of space (Morris et al., 1996; Abrahams et al., 1997; Holdstock et al., 2000; Bohbot et al., 2004; Parslow et al., 2004; Shelton and Gabrieli, 2004; Bartsch et al., 2010). However, other studies (Cave and Squire, 1991; Mayes et al., 1991), including studies using “real-world” tasks designed to emulate spatial memory tasks used in rodents (Bohbot et al., 1998; Bohbot and Corkin, 2007), have produced seemingly inconsistent results suggesting that the hippocampus might not be essential for allocentric spatial learning and memory in humans. Since it is clear that we must use caution when comparing results across human and animal studies using different methodologies (Ravassard et al., 2013; Taube et al., 2013), it is imperative that humans also be tested with paradigms that emulate as closely as possible those used in animals. Studies in which subjects can move about freely in a real-world environment, and therefore perceive and integrate coherent visual, vestibular, proprioceptive, motor efferent copy, somesthetic and auditory information, should be carried out in order to further asses human spatial memory processes (Banta Lavenex et al., 2011; Taube et al., 2013).

Bohbot and colleagues were the first to carry out such studies in which patients with medial temporal lobe lesions were tested in spatial memory tasks designed to emulate tasks commonly used in rodents, such as the Morris water maze (Morris, 1981) and Olton's 8-arm radial maze (Olton and Samuelson, 1976). In their first study, Bohbot and colleagues reported severe spatial memory deficits following unilateral right hippocampal lesion in a number of visuo-spatial memory tasks (Bohbot et al., 1998). In contrast, they reported preserved spatial memory ability in patients with right hippocampal lesion in the Invisible Sensor Task, a dry version of the Morris water maze (Bohbot et al., 1998). Such preservation of spatial memory performance was attributed to the function of the parahippocampal cortex, since patients with right parahippocampal cortex lesions were impaired on this task. Later, Bohbot and Corkin tested patient HM, who had bilateral medial temporal lobe damage which included the rostral hippocampus and entorhinal cortex but spared the caudal hippocampus and the parahippocampal cortex (Corkin et al., 1997; Annese et al., 2014), in the Invisible Sensor Task (Bohbot and Corkin, 2007). Based on HM's performance, they concluded that the hippocampus and the parahippocampal cortex both support allocentric spatial memory, but that the parahippocampal cortex plays a more limited role because HM was able to learn one but not two sensor locations.

### The human hippocampus: in and out of space

In face of the evidence that (1) the hippocampus is implicated in different types of learning and memory processes; (2) experiments on spatial learning sometimes produce seemingly inconsistent findings; and (3) structures outside the hippocampus also process spatial information, some researchers have questioned whether the hippocampus is essential for the processing of allocentric spatial memory in humans. Thus, in order to further investigate the role of the human hippocampus in allocentric spatial learning and memory, we designed a series of experiments to assess the performance of an amnesic patient (P9), with bilateral hippocampal damage following an autoimmune disorder, and 12 age- and sex-matched controls on real-world allocentric spatial memory tasks. In order to evaluate whether deficits following hippocampal damage were specific to the allocentric spatial domain, we also assessed their performance on non-spatial, color memory tasks. We implemented two types of experimental procedures: First, subjects had to learn and memorize information that remained constant over numerous repeated trials; this procedure is akin to a reference memory procedure (Olton et al., 1979). Spatial theories of hippocampal function predict deficits in P9's performance in the spatial condition but not in the color condition; working memory theories of hippocampal function predict preserved performance for P9 in both spatial and color conditions. Second, subjects had to learn and memorize information on a trial-unique basis; this procedure is akin to a working memory procedure (Olton et al., 1979). With the trial-unique procedure, subjects must remember not only which stimuli have been presented as target stimuli, but also when; they must encode the temporal context in which an event occurs. This concept of working memory has been considered by some (Abrahams et al., 1997) as most similar to long-term episodic memory (Tulving, 2002). Episodes that are to be encoded are all unique, since an identical confluence of individual episodic memory components (“what,” the people and the action they are involved in; “where,” the location at which the event takes place; “when,” the time at which the event happens) never repeats. Accordingly, single-trial learning of spatial information can be considered a hallmark of, and a prerequisite for, proper episodic memory function. Spatial theories of hippocampal function predict deficits in P9's performance in the spatial condition but not in the color condition; working memory theories of hippocampal function predict deficits in P9's performance in both color and spatial conditions. As has been shown previously in rodents, monkeys and humans, we found that hippocampal damage is associated with clear deficits in allocentric, spatial relational learning and memory. However, P9's cognitive deficits were not limited to spatial processing. Her performance was also severely impaired when she had to remember trial-unique non-spatial information in the presence of interference between encoding and retrieval, a working memory task. These findings are consistent with the theory that the hippocampus contributes to the integration or binding of multiple items, in order to produce high-resolution/high-capacity representations of personal experience in the service of short-term/working and long-term memory (Yonelinas, 2013).

## Materials and methods

### Participants

Human subjects research was approved by the Intercantonal Ethics Committee for Jura, Neuchâtel, Fribourg (Switzerland), and was in accordance with the NIH guidelines for the use of human subjects in research. Participants gave written informed consent prior to beginning the study.

**Figure 1:**
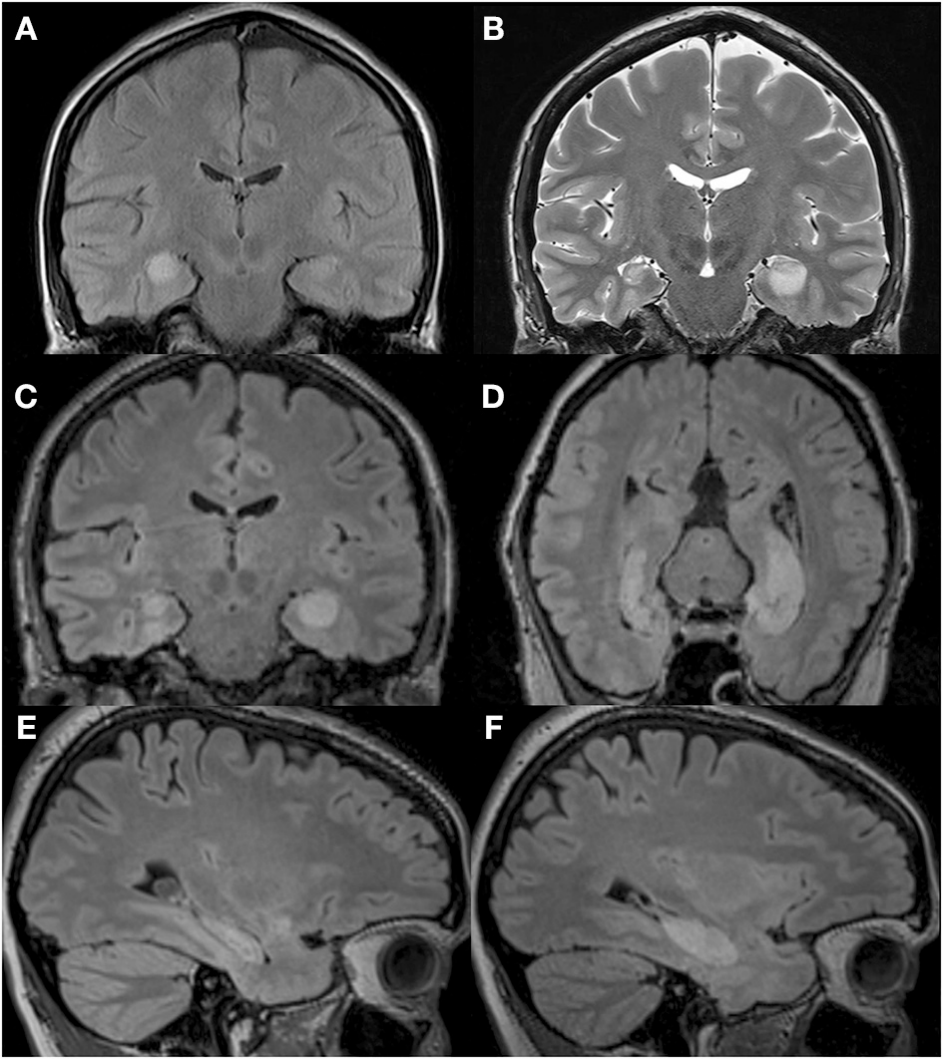
**T2-weighted MRI images of P9's brain showing the extent of the hypersignal reflecting bilateral hippocampal abnormality. (A)** MRI performed upon admittance to the hospital following a generalized tonic-clonic epileptic seizure revealed signs of edema over the right hippocampus. **(B)** MRI performed 2 months following the initial epileptic episode revealed signs of edema over the left hippocampus, and a shrunken right hippocampus. Reproduced from Petel et al. (2011), with permission. **(C–F)** T2-weighted MRI images of P9's brain performed 2.5 months after the initial episode and showing a shrunken right hippocampus in three different planes: coronal **(C)**, parallel to the long axis of the hippocampus **(D)** and sagittal **(E)**, and a hypersignal restricted to the left hippocampus in three different planes: coronal **(C)**, parallel to the long axis of the hippocampus **(D)** and sagittal **(F)**.

**Figure 2:**
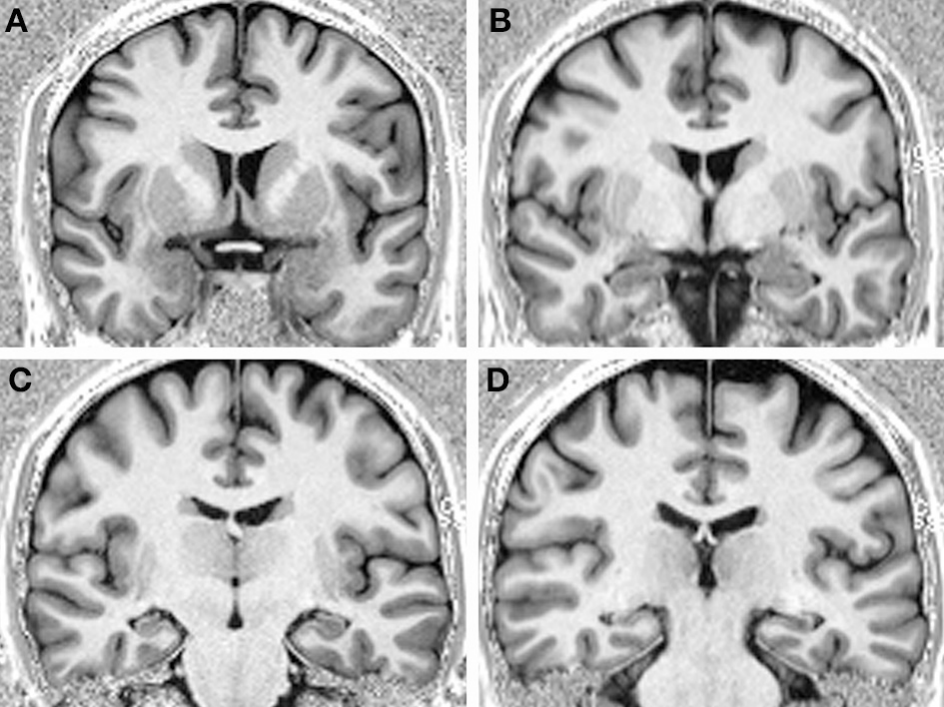
**T1-weighted MRI images of P9's brain performed 3 years after the onset of pathology showing bilateral hippocampal atrophy, and no obvious signs of pathology in the surrounding cortical areas, from rostral (A) to caudal (D)**.

We compared the performance of a profoundly amnesic right-handed patient (P9) (Petel et al., 2011), on a number of allocentric spatial and non-spatial memory tasks, with the performance of 12 healthy, age-matched right-handed female control participants (average biological age: 34.8 years, range 30.1 to 39.4), with similar socio-economical background and level of formal education (P9: 14 years; controls: average: 21, range 13 to 29 years; *t*_(11)_ = 0.38, *p* = 0.711). At the beginning of testing, P9 was 34.5 years of age. One year prior, P9, a married mother of two young children, employed part-time as a medical assistant, and who was normally in good health and had no notable previous medical problems, was admitted to the hospital while experiencing a generalized tonic-clonic epileptic seizure. An EEG showed abnormal elements predominantly over the right temporal lobe. A first MRI revealed an edema over the right hippocampus (Figure 1A) and amygdala, which did not extend into adjacent cortical areas (Petel et al., 2011). Three weeks later, P9's family began noticing that she was having memory problems that seemed to be progressing rapidly. Although subsequent internal and neurological exams were normal, a preliminary neuropsychological exam confirmed severe anterograde verbal and visuo-spatial memory deficits, as well as retrograde deficits for facts and knowledge dating back from 2 to 10 years (Table 1). In contrast, language function, mental calculation abilities, visual perception, motor control and motor planning functions were preserved. Two months following her epileptic episode, two more series of T2-weighted MRIs revealed that although the edema over the right hippocampus was decreasing, the right hippocampus was atrophic (Figures 1B–E). Moreover, the left hippocampus now showed signs of edema (Figures 1B–F). P9 was diagnosed with limbic encephalitis of autoimmune origin, involving antibodies targeting glutamatergic AMPA receptors (Petel et al., 2011). Although detailed examinations of MR images clearly indicate bilateral hippocampal pathology, only a comprehensive post-mortem evaluation of P9's brain could unambiguously determine the absence of subtle alterations of other brain structures (Banta Lavenex et al., 2006; Annese et al., 2014). Nevertheless, experimental work carried out in monkeys has shown that the extent of the edema that can be visualized from the hypersignal that appears following selective ibotenic acid lesion of the hippocampus demonstrates a 95% positive correlation with the extent of the lesion measured on histological preparations of the brain (Malkova et al., 2001). T2-weighted images have also been used to demonstrate focal lesions of the CA1 field of the hippocampus in human transient global amnesia (Bartsch et al., 2010). For P9, T1-weighted structural MRIs performed three years after the onset of pathology showed clear atrophy of both right and left hippocampi, while the surrounding cortical areas (i.e., the entorhinal, perirhinal, and parahippocampal cortices) did not exhibit any obvious signs of pathology (Figures 2A–D).

**Table 1:** **Performance of patient P9 in neuropsychological tests performed 3 months and 18 months after pathological onset**.

	**3 months post-onset**	**18 months post-onset**
**Language**		
Verbal fluency - Animals (1 min)^a^	33/34 (>C50)	
Boston naming test^b^		20 (N)
**Mental calculation abilities**	OK	
**Visual perception**		
Benton facial recognition test^c^	23/27 (N)	
**Motor planning/Motor control**		
Written alternating sequence task (Luria)^d^	N	
Manual alternating sequence task (Luria)^d^	N	
Bimanual coordination	N	
Trail making test - A^e^	19 s, N	
Trail making test - B^e^	32 s, N	
**Anterograde verbal memory**		
10 words (A)	6-8-8 = 22 (C10)	
10 words (C)		6-7-8 = 21 (within limits)
Immediate recognition	6 (SD)	
Delayed recall	1 (SD)	0, 1 FR (SD)
Delayed recognition	4 (SD)	7, 0 FR (SD)
Auditory-verbal learning test^f^		5-5-6-7-7 = 30 (SD)
Immediate recognition		8, 1 FR (SD)
Delayed recall		0 (SD)
Delayed recognition		11, 2 FR (SD)
**Anterograde visuo-spatial memory**		
10 signs	2,5-2-4 = 8.5 (MD)	
15 signs^h^		0-3-5-5.5-4.5 = 18 (SD)
Immediate recognition	8 (C20)	13 (C10)
Delayed recall	0.5 (SD)	2 (SD)
**Retrograde memory**		
Kopelman Autobiographical Memory		
Interview^g^	3/27 (SD)	6/27 (SD)
**Personal semantic memory**		
Childhood	21/21	21/21
Early adult life	19/21	20/21
Recent events	12/21 (within limits)	16/21
**Episodic memory**		
Childhood	1/9 (SD)	2/9 (SD)
Early adult life	1/9 (SD)	3/9 (SD)
Recent events	1/9 (SD)	1/9 (SD)

Abbreviations: FR, false recognition; SD, severe deficit; MD, moderate deficit; N, normal.

a Thuillard and Assal, 1991;

b Thuillard Colombo and Assal, 1992;

c Benton et al., 1983;

d Luria, 1973;

e Roussel and Godefroy, 2008;

f Rey, 1964;

g Kopelman et al., 1989;

h Lanares et al., 1987.

### Testing facilities

**Figure 3:**
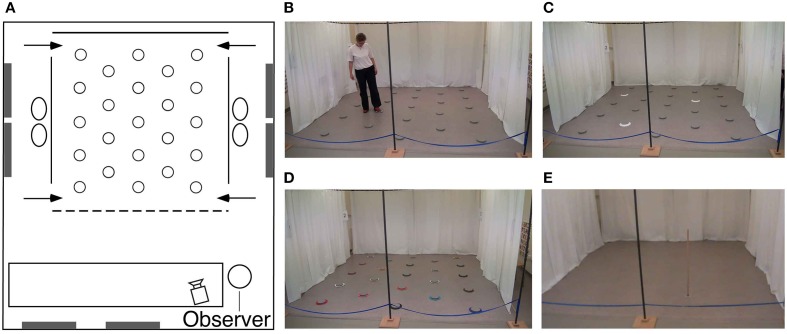
**Testing environment. (A)** Schematic, aerial view of the experimental room (6 × 7 m) containing polarizing features such as doors, windows, tables (white rectangle), chairs, wall posters, etc. Plastic curtains (solid lines) defined three of the boundary walls designating the arena. At each of the four near and far corners of the side walls was a 50 cm gap that served as one of the four different entry points (arrows) through which the subjects must pass in order to enter and exit the testing arena. Twenty-three foot pads were separated by 80 cm from each other and regularly arranged in the arena. **(B)** Picture of the arena with a participant touching an illuminating foot pad. **(C)** Picture of the arena with three cued goal locations during the encoding phase in the repeated-trials location condition. **(D)** Picture of the arena in the color task condition. **(E)** Picture of the arena with the pole used in the find-and-replace experiment. See main text for detailed description of the experimental room and procedures.

All participants were tested at the University of Fribourg in a large room (6 × 7 m; Figure 3) containing many polarizing features such as doors, tables, chairs, curtains, cabinets, wall posters, etc. Within the room was a 4 × 4 m testing arena that consisted of 3 walls made of suspended, opaque plastic curtains (2 m high). Whereas the curtain on the back wall was 4 m wide, the curtains on the side walls extended only 3 m, so that there was a 50 cm gap at the front and the back of the wall, thus creating four entry points through which the participants passed in order to enter and exit the arena. Each entry point was marked by a number (1–4) on the inside and outside walls next to the door. The fourth (front) boundary of the arena was delineated by a rope attached to the two opposing side walls, and suspended 30 cm off the ground. Exterior to the two side walls, the inter-trial waiting area was a corridor (1 × 4 m) that contained two chairs and various visual cues including a door on one side, and different posters, a garbage can and a box of tissues on both sides, none of which could be viewed from within the arena. When participants were not in the arena (i.e., during the inter-phase and inter-trial intervals), they sat in one of the chairs behind one of the two side walls, with their back facing the arena. Importantly, from within the arena, and from the side waiting area, participants had access to distant visual cues within the room. Finally, in order to preclude participants from using an egocentric strategy to solve the task, they entered and exited the arena from different doors on every trial (as instructed by an experimenter during the task).

We conducted two different types of tasks: For the first series, the arena contained 23 regularly arranged, visually identical, gray foot pads (plastic disks, 15 cm in diameter, 2 cm high; Figure 3). Each foot pad was equipped with six LED lights arranged in a circle on the top outer edge of the disk. Foot pads designated as goal locations would illuminate when touched lightly with the foot (Figure 3B). Colored cardboard rings could be placed around the disks in order to make them visually distinct (there were 23 visually-unique rings; Figure 3D). For the second series, the arena was void of all objects except for a wooden pole (a 1.5 m high × 2 cm diameter dowel) with a clear Plexiglas circular base (10 cm in diameter) that served as a foot enabling the pole to stand upright (Figure 3E). The floor of the arena was a uniformly-speckled gray linoleum, and thus could not provide participants with any local landmarks during the task. All testing was videotaped with a video camera located 2.5 m in front of the arena. Both experimenters wore dark sunglasses in order to preclude participants from attending to the eye gaze of the experimenters for clues as to the goal location(s) or color(s).

### Procedures

#### Task 1: repeated-trial learning for 1, 2 or 3 locations

In order to determine whether hippocampal damage affects memory for allocentric spatial information, we tested the ability of P9 and control participants to learn and remember over repeated trials the location(s) of 1, 2, or 3 illuminating foot pad(s) among the 23 foot pads distributed in the arena. Participants were tested on 3 separate days: 1 location on one day, 2 locations on a second day, 3 locations on a 3rd day; tests did not take place on three consecutive days, but were generally one week apart. Participants were given 10 trials to learn the goal location(s), which did not change between trials, but note that different locations were used for the 1, 2, and 3 location tasks. Each trial consisted of two phases: During the first encoding phase, a white ring surrounded each goal location, thus providing a local visual cue as to the goal location (Figure 3C). The participant was instructed to enter the arena through a predetermined door, the number of which an experimenter called out. The participant was required to walk to the goal location and touch it with her foot to illuminate it, after which the disk remained illuminated. As soon as the participant had touched all goal locations, she was instructed to exit the arena by a predetermined door that an experimenter called out and that differed from the door the participant had previously entered through. After a 1-min inter-phase interval, during which an experimenter turned off the lights on the disk(s) and removed the white ring(s), the recall phase began: The participant was instructed to enter the arena through a different predetermined door (nevertheless, on the same side of the arena as the door through which the participant just exited). Now, however, since no local cue(s) surrounded the goal location(s), all goal and decoy locations were visually identical, distinguishable only by their specific location as defined with respect to distal objects in the environment. The participant was asked to show the goal location(s) by walking to it and stepping on the disc to illuminate it, after which it would remain illuminated until the inter-trial interval. A 1-to-2-min inter-trial interval (between the last recall phase and the encoding phase of the next trial) allowed the experimenter to turn off the lights on the disk(s) and replace the white ring(s) in preparation for the next trial. The same procedure repeated for 10 trials within a daily session.

#### Task 2: repeated-trial learning for 1, 2, or 3 colors

In order to determine whether hippocampal damage affects memory for non-spatial information, we tested the ability of P9 and control participants to learn and remember over repeated trials the color(s) of 1, 2, or 3 ring(s) surrounding the illuminating foot pad(s). Each of the 23 foot pads in the arena was surrounded by a uniquely colored cardboard ring (Figure 3D). As before, participants were tested on 3 separate days: 1 color on one day, 2 colors on a 2nd day, 3 colors on a 3rd day. Participants were given 10 trials to learn the goal color(s), which did not change between trials (but note that different colors were used for the 1, 2, and 3 goal tasks). During the encoding phase, the goal disks were already illuminated when the participant entered the arena. The participant was required to walk to each illuminated disk and step on it. During the 1-min inter-phase interval, an experimenter turned off the lights on the disk(s) and moved the illuminating disk(s) and colored ring(s) to other locations in the arena, thus making location irrelevant and unreliable. During the recall phase, the participant was asked to show the goal color(s) by locating the correct ring and stepping on its associated disk, after which the disk would remain illuminated. A 1-to-2-min inter-trial interval allowed an experimenter to rearrange the colored disks and their associated illuminating foot pads for the next trial. For each of the three memory loads (1, 2, or 3), a total of 6 colored rings would be moved during both the inter-phase and inter-trial interval, although the color of the goal(s) would never change. The same procedure repeated for 10 trials within a daily session.

#### Task 3: trial-unique learning for 1, 2 or 3 locations

In order to determine whether hippocampal damage affects working memory for allocentric spatial information, and to emulate episodic-like memory conditions, we tested the ability of P9 and control participants to learn and remember the location(s) of 1, 2, or 3 illuminating foot pad(s) on a trial-unique basis, i.e., goal locations would change between trials. Participants were tested on 3 separate days, after a 15-min break following the repeated-trials location task with the same number of goals (Task 1). Each trial consisted of two phases: During the encoding phase, no visual cues marked the goal location(s), and thus to discover the new goal location(s) participants had to explore the arena, touching the disks in order to identify the location of the illuminating disk(s). The disks would immediately extinguish once participants removed their foot. During the 1-min inter-phase interval, participants were required to count backward by 3 from a predetermined number (1000 in trial 1, 899 in trial 2, 798 in trial 3, 697 in trial 4, 596 in trial 5, 495 in trial 6, 394 in trial 7, 999 in trial 8, 898 in trial 9, and 797 in trial 10). Although the experimenters did not control the accuracy with which the participant counted backward, they listened for fluency and prompted the participant to continue counting if any pause was evident. During the recall phase, participants were asked to show the location of the goal(s) by walking to it and stepping on the disk to illuminate it. The disk did not remain illuminated once the participant removed their foot from it. A 1-to-2-min inter-trial interval allowed an experimenter to rearrange the illuminating foot pads into the next trial-unique array. The same procedure repeated for 10 trials within a daily session. For P9, but not for the control participants, we repeated this trial-unique spatial task 13 months later. This time, however, we did not introduce inter-phase interference; P9 was not asked to count backward during the 1-min inter-phase interval.

#### Task 4: trial-unique learning for 1, 2, or 3 colors

In order to determine whether hippocampal damage affects working memory for non-spatial information under episodic-like memory conditions, we tested the ability of P9 and control participants to learn and remember the color(s) of 1, 2, or 3 ring(s) surrounding the illuminating foot pads on a trial-unique basis, i.e., goal colors would change between trials. Participants were tested on 3 separate days, after a 15-min break following the repeated-trials color task with the same number of goals (Task 2). Each trial consisted of two phases: During the encoding phase, no visual cue marked the goal color(s) (the disks were not illuminated), and thus to discover the goal color(s) participants had to explore the arena, touching the disks in order to identify the color(s) surrounding the illuminating disk(s). The disks would immediately extinguish once participants removed their foot. During the 1-min inter-phase interval, while an experimenter moved the colored rings and their associated illuminating disks, participants were required to count backward by 3 from a predetermined number as described in Section Task 3: Trial-Unique Learning for 1, 2, or 3 Locations. During the recall phase, participants were asked to show the color(s) surrounding the goal(s) by walking to it and stepping on the disk to illuminate it. A 1-to-2-min inter-trial interval allowed an experimenter to associate new colored disks with the illuminating foot pads and place them in new locations for the next trial-unique array. The same procedure repeated for 10 trials within a daily session. For P9, but not for the control participants, we repeated this trial-unique color task 13 months later. This time, however, we did not introduce inter-phase interference; P9 was not asked to count backward during the 1-min inter-phase interval.

#### Task 5: find-and-replace: repeated-trial learning with 1 location

In order to determine how hippocampal damage impacts precision coding for one location, we tested the ability of P9 and control participants to learn and remember over repeated trials the location of one object, an upright 1.5 m-high pole, placed in the otherwise empty arena. During the encoding phase, the participant would enter the arena through a predetermined door and find the pole standing at a predetermined location. The participant was required to go to the pole and pick it up. The participant was immediately instructed to exit the arena by a predetermined door that an experimenter called out. After a 1-min inter-phase interval without any interference (i.e., no instructions to count backward), the recall phase began: The participant entered through another predetermined door (not the same through which she just exited, but on the same side of the arena), and was required to place the pole in the exact same location in which she found it previously. A 1-to-2-min inter-trial interval (between the last recall phase and the encoding phase of the next trial) allowed the experimenters to use a laser tape measurer to determine the exact position of the pole in the arena, as the participant placed it, and replace the pole in its standard location for the next trial. The same procedure repeated for a total of 10 trials.

#### Task 6: find-and-replace: trial-unique learning with 1 location

In order to determine how hippocampal damage impacts precision coding for one location on a trial-unique basis, emulating working memory and episodic-like memory conditions, we tested the ability of P9 and control participants to learn and remember the location of the upright pole, placed in the otherwise empty arena, on a trial-unique basis. As described above, participants were required to retrieve the pole, and after a 1-min inter-phase interval without interference, put the pole back exactly where they had found it. There were three predetermined goal locations within each quadrant of the arena (for a total of 12 trial-unique locations), and the pseudo-randomly chosen order of the locations was predetermined before testing began with the restriction that the pole could not be placed in the same quadrant on two consecutive trials. A 1-to-2-min inter-trial interval allowed the experimenters to use a laser tape measurer to determine the exact position of the pole in the arena, as the participant placed it, and place the pole at the next predetermined location within the arena. The same procedure repeated for a total of 12 trials.

### Data analysis

Because the latency to solve a task might be influenced by different factors such as confidence, strategy, and motivation, we do not rely on latency as a measure of spatial memory ability. Instead, we determine whether subjects are accurate at recalling the goal locations by determining whether and how well they discriminate goal locations from non-goal locations, thus demonstrating that subjects do or do not remember where the goals were. The following measures were used to describe and analyze the subjects' behavior and performance: (1) The number of goal locations visited before making an error (i.e., visiting a non-goal location); (2) the number of errorless trials; (3) the number of trials in which the first location visited was a goal location; (4) the types of locations visited when subjects made an error in their first choice upon entering the arena (i.e., a previous goal location, a location adjacent to a current goal location, or another location). We used Crawford and Howell's modified *t*-test procedure to compare P9's performance with that of the control participants (Crawford and Howell, 1998). We used repeated measures General Linear Model (GLM) analyses and paired *t*-tests to compare P9's performance, or that of controls, between experimental conditions.

## Results

### Number of correct choices before the first error (CBE)

**Figure 4:**
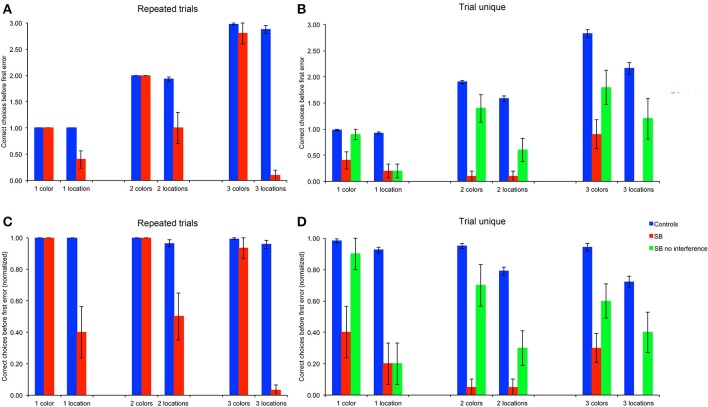
**Number of correct choices before the first error (CBE): Number of correct choices before making an error. (A,B)** Number of correct choices, absolute average numbers per trial in the repeated-trials and trial-unique conditions. **(C,D)** Normalized number of correct choices per trial in the repeated-trials and trial-unique conditions: CBE was divided by the number of goal locations. P9's performance was impaired as compared to controls in all conditions, except for the color, repeated-trials condition with one and two goals, and in the trial-unique color condition with one goal and no interference.

We first considered the number of goal locations subjects visited before making an error, as a measure to estimate memory capacity (Figures 4A,B). To enable statistical comparison between memory loads, subjects' average CBE across 10 trials were normalized by dividing the CBE by the number of goals for each memory load, i.e., 1, 2, or 3, respectively (Figures 4C,D).

#### Repeated-trials

P9's CBE did not differ from that of controls in the color conditions with one or two colors, but it was lower than controls' with three colors [Figures 4A,C: *t*_(11)_ = 2.70, *p* < 0.021]. In contrast, P9's CBE was consistently lower than controls' in the allocentric spatial conditions with one [*t*_(11)_ = 5.76, *p* < 0.0002], two [*t*_(11)_ = 6.24, *p* < 0.0001] or three [*t*_(11)_ = 10.55, *p* < 0.0001) locations.

#### Trial-unique

P9's CBE was lower than controls' in all conditions with the trial-unique procedure [Figures 4B,D: all *t*_(11)_ > 3.93, *p* < 0.003]; except in the trial-unique condition with one color, when the performance of P9 without an interference task (Figure 4: green bars) was compared with that of controls performing an interfering task [Figure 4: blue bars; *t*_(11)_ = 2.05, *p* = 0.064]. However, P9's CBE was lower than controls' when she performed an interference task in the trial-unique, one color condition (Figure 4: red bars: *t*_(11)_ = 14.39, *p* < 0.0001].

#### Repeated-trials vs. trial-unique

Overall, the CBE of control subjects differed based on the type of trials [*F*_(1, 66)_ = 61.340, *p* < 0.0001; repeated-trials > trial-unique with interference), the testing condition [*F*_(1, 66)_ = 79.788, *p* < 0.0001; color > location] and the memory load [1, 2, or 3 goals; *F*_(2, 66)_ = 20.842, *p* < 0.0001]; moreover, there were significant interactions between all factors (all *p* < 0.005). In the color condition, controls' CBE was higher in the repeated-trials condition than in the trial-unique condition with interference [*F*_(1, 33)_ = 7.436, *p* = 0.01], but it did not differ between memory loads [*F*_(2, 33)_ = 2.258, *p* = 0.120]. In the spatial condition, CBE was higher in the repeated-trials condition [*F*_(1, 33)_ = 55.105, *p* < 0.0001]; it differed between memory loads in the trial-unique condition [*F*_(2, 33)_ = 14.532, *p* < 0.0001; one > two > three), but not in the repeated-trials condition [*F*_(2, 33)_ = 1.662, *p* = 0.205].

P9's CBE also differed based on the type of trials [*F*_(2, 108)_ = 36.571, *p* < 0.0001; repeated-trials vs. trial-unique without interference vs. trial-unique with interference], the testing condition [*F*_(1, 54)_ = 71.443, *p* < 0.0001; color > location), and the memory load [*F*_(2, 54)_ = 3.649, *p* = 0.033; one > three]; there was a significant interaction between the type of trials and the testing condition [*F*_(2, 108)_ = 10.069, *p* < 0.0001]. In the color condition, P9's CBE was higher in the repeated-trials condition [*F*_(2, 54)_ = 52.410, *p* < 0.0001], as compared with the trial-unique conditions with and without interference; CBE was also higher in the trial-unique condition without interference than in the trial-unique condition with interference (all *p* < 0.05). In the spatial condition, P9's CBE was lower in the trial-unique condition with interference than in the other two conditions, which did not differ from each other [*F*_(2, 54)_ = 3.765, *p* < 0.0001].

In sum, based on the number of goals subjects visited before making an error (CBE), a measure thought to reflect memory capacity, P9 was impaired in: (1) allocentric, spatial relational learning over repeated trials, irrespective of memory load; (2) trial-unique, allocentric spatial relational learning, irrespective of memory load and interference conditions; (3) trial-unique, color learning, irrespective of memory load and interference conditions, although the impairment was greater with interference. In contrast, P9 was not impaired in repeated-trial color learning for 1 or 2 colors.

### Number of errorless trials (NET)

**Figure 5:**
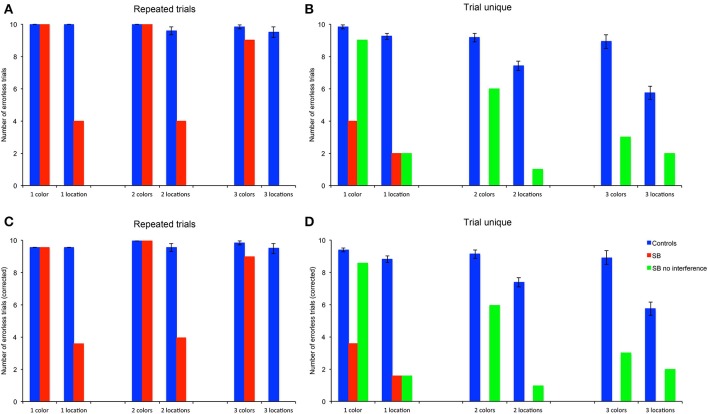
**Number of errorless trials (NET): number of trials in which subjects made no errors. (A,B)** Number of trials. **(C,D)** Number of trials corrected for the probability to find all goal locations by chance (4.3478% with one location (1/23), 0.3953% with 2 locations (2/23 ^*^ 1/22), 0.0565% with 3 locations (3/23 ^*^ 2/22 ^*^ 1/21). P9's performance was impaired as compared to controls in all conditions, except for the color, repeated-trials condition with one, two and three goals, and in the trial-unique color condition with one goal and no interference.

Because CBE is calculated by averaging over trials, it does not give an indication as to whether P9 or control subjects exhibit perfect memory performance on some trials. We thus determined the number of trials in which subjects made no error, as a measure of perfect memory performance (Figures 5A,B). To enable statistical comparison between memory loads, we corrected the number of errorless trials by subtracting the probability to make errorless trials by chance in the different conditions (Figures 5C,D): 0.43478 with one goal (1/23^*^10), 0.03953 with two goals (2/23^*^1/22^*^10), and 0.00565 with three goals (3/23^*^2/22^*^1/21^*^10).

#### Repeated-trials

P9's NET did not differ from controls' in the color condition. For one and two colors, P9 and controls performed errorlessly; for three colors P9's NET did not differ significantly from controls [*t*_(11)_ = 2.05, *p* = 0.064]. In contrast, P9's NET was consistently lower than controls' in the allocentric spatial conditions with one [*t*_(11)_ = 5.76, *p* < 0.0002], two [*t*_(11)_ = 6.76, *p* < 0.0001] or three [*t*_(11)_ = 9.12, *p* < 0.0001] locations.

#### Trial-unique

P9's NET was lower than controls' in all conditions with the trial-unique procedure [Figures 5B,D: all *t*_(11)_ > 3.93, *p* < 0.003]; except in the trial-unique one color condition with no interference, where the difference with controls performing an interfering task just failed to reach significance [*t*_(11)_ = 2.05, *p* = 0.064].

#### Repeated-trials vs. trial-unique

Overall, controls' NET differed based on the type of trials [*F*_(1, 66)_ = 107.557, *p* < 0.0001; repeated-trials > trial-unique with interference], the testing condition [*F*_(1, 66)_ = 75.734, *p* < 0.0001; color > location] and the memory load [1, 2, or 3 goals; *F*_(2, 66)_ = 17.073, *p* < 0.0001]; there were significant interactions between all the factors (all *p* < 0.005). In the color condition, controls' NET was higher in the repeated-trials condition than in the trial-unique condition with interference [*F*_(1, 33)_ = 17.165, *p* < 0.0001], and did not differ between memory loads [*F*_(2, 33)_ = 0.416, *p* = 0.663]. In the spatial condition, controls' NET was higher in the repeated-trials condition [*F*_(1, 33)_ = 94.370, *p* < 0.0001]; it differed between memory loads in the trial-unique condition [*F*_(2, 33)_ = 29.933, *p* < 0.0001; one > two > three], but not in the repeated-trials condition [*F*_(2, 33)_ = 0.037, *p* = 0.964].

P9's NET also differed based on the type of trials [*F*_(2, 108)_ = 32.698, *p* < 0.0001; repeated-trials > trial-unique without interference > trial-unique with interference], the testing condition [*F*_(1, 54)_ = 65.528, *p* < 0.0001; color > location), and the memory load [*F*_(2, 54)_ = 7.960, *p* = 0.001; one > two = three]; there was a significant interaction between the type of trials and the testing condition [*F*_(2, 108)_ = 12.347, *p* < 0.0001]. In the color condition, P9's NET was higher in the repeated-trials condition [*F*_(2, 54)_ = 47.100, *p* < 0.0001], as compared with the trial-unique conditions with and without interference; NET was also higher in the trial-unique condition without interference than in the trial-unique condition with interference (all *p* < 0.05). In addition, P9's NET varied with memory load [*F*_(2, 27)_ = 8.008, *p* = 0.002; one > two = three]. In the spatial condition, P9's NET did not differ based on the testing conditions [*F*_(2, 54)_ = 2.229, *p* = 0.117], or memory load [*F*_(1, 27)_ = 1.591, *p* = 0.222].

In sum, based on the number of errorless trials (NET), a measure reflecting perfect memory performance, P9 was impaired in: (1) allocentric, spatial relational learning over repeated trials, irrespective of memory load; (2) trial-unique, allocentric spatial relational learning, irrespective of memory load and interference conditions; (3) trial-unique, color learning, irrespective of memory load and interference conditions, although the impairment was greater with interference. In contrast, P9 was not impaired in repeated-trial color learning for 1, 2, or 3 colors.

### Number of trials with the first choice correct (FCC)

**Figure 6:**
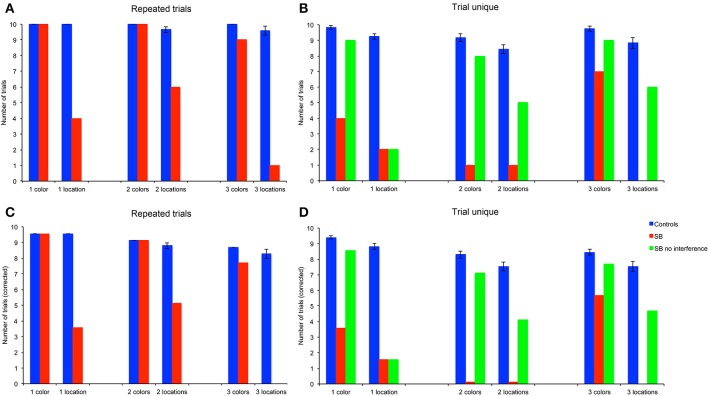
**Number of trials with the first choice correct (FCC). (A,B)** Number of trials. **(C,D)** Number of trials corrected for the probability to find the first goal location by chance (1/23 for one location, 2/23 for two locations, 3/23 for three locations). P9's performance was impaired as compared to controls in all conditions, except for the color, repeated-trials conditions with one, two and three goals, and in the trial-unique color conditions with one, two and three goals and no interference.

We also analyzed the number of trials in which subjects chose a goal as their first choice upon entering the arena (Figures 6A,B), in order to determine whether P9 demonstrated residual memory capacities that would not be evidenced by the more stringent measures of performance described above. In order to compare the part of the performance that can be unambiguously attributed to memory, subjects' average FCC across 10 trials were corrected by subtracting the probability of finding the first goal by chance with different memory loads, i.e., 1/23, 2/23, or 3/23, respectively (Figures 6C,D).

#### Repeated-trials

P9's FCC did not differ from controls' in the color conditions. For one and two colors P9 and controls performed errorlessly; for three colors P9's FCC was not significantly lower than controls'. In contrast, P9's FCC was consistently lower than controls' in the allocentric spatial conditions with one [*t*_(11)_ = 57.64, *p* < 0.0001], two [*t*_(11)_ = 5.40, *p* = 0.0002] or three [*t*_(11)_ = 8.27, *p* < 0.0001] locations.

#### Trial-unique

P9's FCC was lower than controls' in all conditions with the trial-unique procedure with interference [Figure 6D: all *t*_(11)_ > 2.28, *p* < 0.05]. In absence of interference in the spatial condition, P9's FCC was lower than controls' performance with interference for all memory loads [one location, *t*_(11)_ = 11.20, *p* < 0.0001; two locations, *t*_(11)_ = 3.29, *p* < 0.001; three locations, *t*_(11)_ = 2.28, *p* < 0.05]. In contrast, FCC did not differ between controls tested with an interference procedure and P9 tested without interference in the color condition [one color, *t*_(11)_ = 2.05, *p* = 0.064; two colors, *t*_(11)_ = 1.34, *p* = 0.20; three colors, *t*_(11)_ = 1.15, *p* = 0.27].

#### Repeated-trials vs. trial-unique

Overall, controls' FCC differed based on the type of trials [*F*_(1, 66)_ = 30.907, *p* < 0.0001; repeated-trials > trial-unique with interference], the testing condition [*F*_(1, 66)_ = 22.994, *p* < 0.0001; color > location] and the memory load [1, 2, or 3 goals; *F*_(2, 66)_ = 41.841, *p* < 0.0001]; there was a significant interaction between the type of trials and testing conditions [*F*_(1, 66)_ = 4.346, *p* = 0.041]. In the color condition, controls' FCC was higher in the repeated-trials condition than in the trial-unique condition with interference [*F*_(1, 33)_ = 15.184, *p* < 0.0001]; it differed between memory loads in the trial-unique condition [*F*_(2, 33)_ = 10.414, *p* < 0.0001; one > two = three], but not in the repeated-trials condition since performance was errorless for the three memory loads. In the spatial condition, controls' FCC was higher in the repeated-trials condition [*F*_(1, 33)_ = 18.233, *p* < 0.0001]; it differed between memory loads in both the repeated-trials [*F*_(2, 33)_ = 10.643, *p* < 0.0001; one > two = three] and trial-unique [*F*_(2, 33)_ = 6.984, *p* = 0.003; one > two > three] conditions.

P9's FCC also differed based on the type of trials [*F*_(2, 108)_ = 21.464, *p* < 0.0001; repeated-trials = trial-unique without interference > trial-unique with interference], the testing condition [*F*_(1, 54)_ = 63.158, *p* < 0.0001; color > location], but not the memory load [*F*_(2, 54)_ = 0.536, *p* = 0.588]; there was a significant interaction between the type of trials and memory load [*F*_(4, 108)_ = 3.250, *p* = 0.015]. In the color condition, P9's FCC was lower in the trial-unique condition with interference and did not differ between the repeated-trials and the trial-unique without interference conditions [*F*_(2, 54)_ = 20.583, *p* < 0.0001]. In the spatial condition, this effect was similarly observed for two [*F*_(2, 18)_ = 4.846, *p* = 0.021] or three [*F*_(2, 18)_ = 7.154, *p* = 0.005] locations, but not for one location [*F*_(2, 18)_ = 0.545, *p* = 0.589].

In sum, based on the number of first correct choices upon entering the arena (FCC), P9 was impaired in: (1) allocentric, spatial relational learning over repeated trials, irrespective of memory load; (2) trial-unique, allocentric spatial relational learning, irrespective of memory load and interference conditions; (3) trial-unique, color learning, irrespective of memory load, but only in the tasks with interference. In contrast, P9 was not impaired in repeated-trial color learning for 1, 2, or 3 colors.

### Analysis of errors: spatial versus temporal resolution

**Figure 7:**
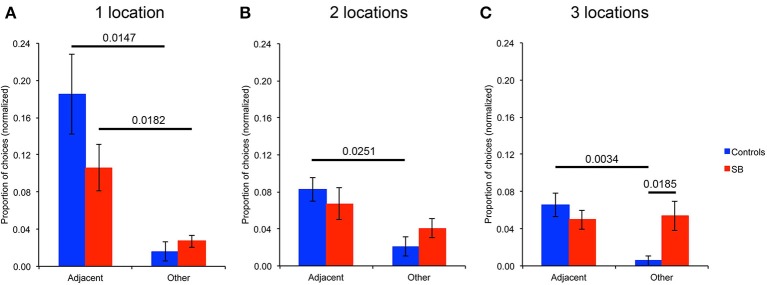
**Analysis of errors: spatial versus temporal resolution**. Types of locations visited when making an error on the first choice upon entering the arena. **(A)** Analysis for the tasks with one goal location. **(B)** Analysis for the tasks with two goal locations. **(C)** Analysis for the tasks with three goal locations. Note that the number of choices of different locations is normalized based on the probability to make that choice.

In order to further characterize P9's behavior when she did not perform optimally in the spatial conditions, we analyzed the types of locations chosen when she made an error on her first choice upon entering the arena (Figure 7). Specifically, we analyzed whether incorrect first choices made with a memory load of one, two, or three locations (across spatial conditions, including repeated-trials and trial-unique conditions with and without interference) corresponded to: (1) The previous goal location(s), thus representing a difficulty in distinguishing between different trials, i.e., a temporal resolution error; (2) locations immediately adjacent to the goal location(s), thus representing a difficulty in distinguishing close locations, i.e., a spatial resolution error; (3) other unrelated, random locations. Note that the numbers of different types of choices were normalized based on the probability to make those choices. For example, with a memory load of three, the number of choices of a previous location was divided by three. The number of locations immediately adjacent to the goal locations varied by location, and thus was determined for every subject's individual trial in which she made an error in her first choice. For example, with one goal location located in the middle of the arena (Figure 3A), there were six adjacent locations, so the choice of an adjacent location was divided by six (i.e., 1/6). Similarly, for every other location in the arena, the probability of choosing that location was specifically determined.

With one goal location (Figure 7A), P9 chose significantly more locations adjacent to the goal than other unrelated locations when she made an error on her first choice upon entering the arena (*p* = 0.0182). This behavior was not significantly different from that of controls who also chose more adjacent locations than other unrelated locations (*p* = 0.0147). P9 chose the previous goal location only once across all trial-unique trials with one goal location; controls never chose the previous goal location. With two goal locations (Figure 7B), controls chose more locations adjacent to the goals (*p* = 0.0251), whereas P9 did not (*p* = 0.3441). P9 chose one of the two previous goal locations only once across all trial-unique trials with two goal locations; controls never chose a previous goal location. Similarly, with three goal locations (Figure 7C), controls chose more locations adjacent to the goals (*p* = 0.0034), whereas P9 did not (*p* = 0.8545). Again, P9 chose only twice one of the three previous goal locations across all trials with three goal locations. One time, one control subject chose a previous goal location when there were three goal locations.

In sum, when P9 made an error in her first choice upon entering the arena in the spatial conditions with one goal location, she did not choose randomly, but instead chose preferentially locations adjacent to the goal. This was not the case with two and three goal locations. Thus, although a number of other measures revealed that P9 was severely impaired, as compared to controls, in performing an allocentric, spatial relational learning and memory task with one, two or three locations, her behavior was not random as she was able to acquire, and demonstrate, some sort of knowledge about one, but not two or three location(s) in the open-field arena.

### Find-and-replace task

In order to characterize the precision of the spatial representation enabling P9 to choose locations adjacent to the goal location, and yet clearly insufficient to perform as well as controls in a task with well-defined visible goal and decoy locations 80 cm apart, we designed another task in which P9 and control subjects had to learn and remember the location of a single object located in the otherwise empty arena (Figure 3E). This task was performed following two different procedures: a repeated-trial procedure in which the goal location remained the same for 10 trials, and a trial-unique procedure in which the goal location changed between trials.

#### Repeated-trials

**Figure 8:**
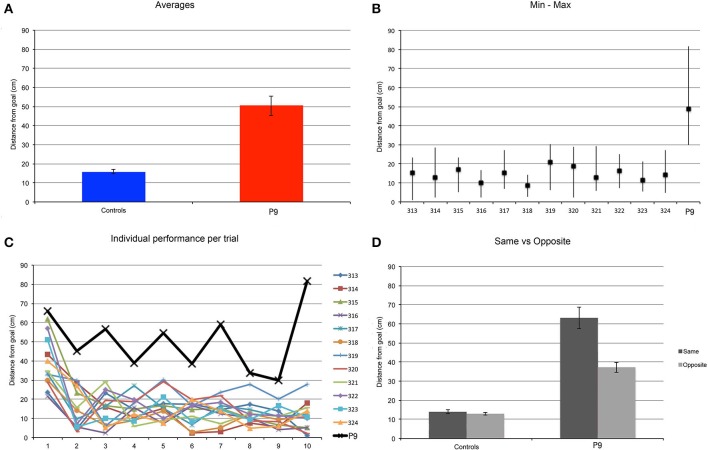
**Find and Replace: the same location was used for 10 repeated trials**. P9's performance was impaired as compared to controls. **(A)** Average distance to the actual goal location. P9 > Controls, *t*_(11)_ = 9.776, *p* < 0.0001. **(B)** Average, minimum and maximal values for subject P9 and 12 age-matched controls. Note that data from the first trial are not included in this analysis. **(C)** Individual subjects' performance per trial. **(D)** Average distance to the goal, as a function of the starting location in the arena in the replace phase. The distance to the goal (spatial error) was greater when P9 entered the arena on the same side where the goal was located, as compared to when she entered from the opposite side of the arena. Performance of control subjects did not vary based on the position of the entrance relative to the goal.

P9 was less precise than controls when replacing the object in the arena [*t*_(11)_ = 9.776, *p* < 0.0001; Figure 8A]. Control subjects replaced the object at an average distance to the goal location of 16 cm, whereas P9's average distance to the goal location was 50 cm; that is about 3 times the distance of controls. Excluding the performance on the first trial, we found that P9's smallest distance to the goal location on any given trial (trial 9: 29.76 cm) was essentially the same as the controls' greatest distance to the goal location on any given trial (subject 319, trial 5: 30.28 cm; Figure 8B). We also found that controls' performance improved after the first trial and stayed at the same level for the remaining trials [*F*_(9, 99)_ = 13.528, *p* < 0.0001; trial 1 > trials 2–10, all *p* < 0.0001]. P9's performance was not different from controls' for the first trial, but was significantly lower for all the other trials (trials 2–10; all *p* < 0.05). In contrast to controls, P9's performance did not improve, varied greatly and seemingly fluctuated in precision between odd and even trials (Figure 8C). Detailed analyses revealed that P9's performance was dependent on the entrance used during the replace phase, and thus her starting position relative to the goal when entering the arena. P9's performance was worse when she entered the arena through a door located on the same side of the goal, as compared to when she entered the arena through a door located on the opposite side of the arena (*p* = 0.0044; Figure 8D). Controls' performance did not differ based on the door used to enter the arena during the replace phase (*p* = 0.4532).

#### Trial-unique

**Figure 9:**
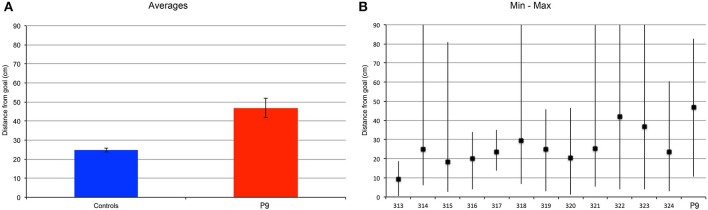
**Find and replace: a different location was used for each of the 12 different trials**. P9's performance was impaired as compared to controls. **(A)** Average distance to the actual goal location. P9 > Controls, *t*_(11)_ = 2.503, *p* = 0.0293. Note that for controls, the distance from the goal was greater in the trial-unique condition, as compared to the repeated trial condition (Figure 8), *p* = 0.0037. P9's performance did not differ between repeated-trials and trial-unique conditions, *p* = 0.6656. **(B)** Average, minimum and maximal values for subject P9 and 12 age-matched controls.

We found similar results when subjects were tested with a trial-unique procedure and the goal location changed between trials (Figure 9). P9 was less precise than controls when replacing the object in the arena [*t*_(11)_ = 2.503, *p* = 0.0293]. Controls replaced the object at an average distance of about 25 cm, whereas P9's average distance to the goal location was about 47 cm; that is about 2 times the distance of controls. Note that for controls, the distance from the goal was greater in the trial-unique condition than in the repeated-trials condition (*p* = 0.0037), whereas P9's performance did not differ between repeated-trials and trial-unique conditions (*p* = 0.6656). Interestingly, controls' performance varied greatly between trials, with some subjects occasionally replacing the object in a completely different quadrant of the arena on some of the trial-unique trials (Figure 9B); this behavior was never observed in the repeated-trials condition (Figure 8B). Despite these errors, the average performance of controls remained significantly better than P9's (Figure 9A).

In sum, in these find-and-replace tasks, although P9 was in general able to place the object in the proper quadrant of the arena, her spatial precision was two to three times lower than that of controls depending on the specific testing conditions.

## Discussion

We have found that hippocampal damage in an amnesic patient, P9, is associated with clear deficits in allocentric, spatial relational learning and memory. However, P9's cognitive deficits were not limited to spatial processing. Her performance was also severely impaired when she had to remember trial-unique color information in the presence of interference between encoding and retrieval, a working memory task. Here, we compare our findings with those of previous studies of spatial and working memory in humans. We conclude that, altogether, these findings are consistent with the theory that the hippocampus contributes to the integration or binding of multiple items, in order to produce high-resolution/high-capacity representations of spatial and non-spatial information in the service of short-term/working and long-term memory (Yonelinas, 2013).

### Comparison with previous studies of human spatial memory

Given the extent of the literature establishing the role of the rodent hippocampus in allocentric spatial learning and memory (Morris, 2007), and the importance of testing humans with paradigms that emulate as closely as possible those used in animals (Taube et al., 2013), we discuss primarily studies that investigated the role of the human hippocampus in real-world situations. These studies are few, yet they are fundamental in order to evaluate possible interspecies differences in spatial information processing. Indeed, in all species the hippocampus is at the apex of a hierarchy of associational networks, which ultimately integrates much of the processing that takes place within the neocortex and a number of subcortical brain areas (Lavenex and Amaral, 2000). In normal circumstances, information derived from different sensory modalities is coherent and fully integrated by the brain, including the hippocampus, to elaborate consistent representations of personal experience. In contrast, it is typically the case in virtual reality studies that different inputs derived from different sensory modalities are inconsistent, so that both cooperative and competitive interactions between sensory cues contribute to regulate hippocampal place cell activity thought to underlie the formation of a rat's cognitive map (Ravassard et al., 2013). Accordingly, in the case of a person lying in an MRI scanner or sitting in front of a computer screen, vestibular, proprioceptive and tactile information are all coherently coding the absence of movement of the individual, whereas visual information is typically used to make the person believe that s/he is actively or passively moving while exploring the virtual environment. Although one might argue that humans are very much accustomed to such discrepancies due to their use of modern modes of transportation, one cannot ignore the fact that these conditions are fundamentally different from those experienced by animals, especially rodents, tested in the laboratory (Banta Lavenex and Lavenex, 2010; Lavenex and Banta Lavenex, 2013; Ravassard et al., 2013; Taube et al., 2013). Accordingly, we have previously shown that spatial memory performance is not the same when changes in viewpoint are produced by moving a display in front of a stationary subject as compared to when the changes are produced by the movement of the subject around a stationary display (Banta Lavenex et al., 2011). Nevertheless, previous studies carried out in humans with tabletop displays, computer screens and virtual reality paradigms are largely in agreement with findings in non-human animals, and affirm the central role of the human hippocampus in allocentric spatial coding. Curiously, however, the few real-world laboratory experiments carried out to date to investigate allocentric spatial coding in humans have raised uncertainty regarding the role of the human hippocampus in allocentric spatial memory.

#### The Invisible Sensor Task (IST)

Bohbot and colleagues designed a task analogous to the Morris water maze in which human subjects were asked to look for an invisible weight sensor hidden underneath a carpet covering the floor of a small trapezoid room (approximately 9–10 m^2^), not controlled for the presence of objects and visual cues at the periphery (Bohbot et al., 1998, 2002). In this task, patients with unilateral, right or left hippocampal lesion sparing the parahippocampal cortex were seemingly unimpaired with a 30 min delay, whereas patients with lesions to the right parahippocampal cortex were impaired. Bohbot and colleagues interpreted these results as evidence against the hypotheses that the hippocampus is critical for allocentric spatial memory and the parahippocampal cortex critical for egocentric spatial memory (Bohbot et al., 2000). Similarly, patient HM also exhibited relative success on the IST when he was tested with one single goal location (Bohbot and Corkin, 2007), despite bilateral medial temporal damage including the rostral half of the hippocampus and the entorhinal cortex, but preserving the caudal portion of the hippocampus and the parahippocampal cortex (Corkin et al., 1997; Annese et al., 2014). Bohbot and Corkin therefore concluded that the hippocampus and the parahippocampal cortex both support allocentric spatial memory, but that the parahippocampal cortex plays a limited role because HM was able to learn one but not two sensor locations. We do not believe this interpretation to be correct. Instead, a close examination of the specific representational demands of the IST suggests that the spatial deficits observed following lesion of the parahippocampal cortex might also be explained by a deficit in egocentric coding, since subjects started the recall trial after a 30 min delay from the same area of the room where they started the search trial (Bohbot et al., 1998), and HM started from roughly the same start location on many trials (Bohbot and Corkin, 2007). Accordingly, the apparent sparing of allocentric capacities in patients with hippocampal lesions and intact parahippocampal cortices might be due to a preserved ability to make use of a viewpoint-dependent memory of the goal location. In contrast to what was suggested elsewhere by the authors (Bohbot et al., 2000), the IST with only one goal location, one or two starting points, and environmental cues that can be perceived and directly aligned with the goal location, might not be sufficient to preclude the reliance on viewpoint-specific snapshot memories of the room from the goal or starting locations: i.e., in relation to the view of a single scene (Bohbot et al., 2004).

The alternative interpretation suggesting that the parahippocampal cortex enables hippocampus-lesioned subjects to remember one location by matching it with a remembered viewpoint was also discussed by Bohbot et al. (2004), Bohbot and Corkin (2007), and is supported by our current findings with P9. Indeed, although P9 was clearly impaired in all allocentric spatial memory tasks, her behavior was not random and she was clearly able to determine the general area of a single goal within a square, 4 × 4 m, open-field arena surrounded by curtains on three sides, but with much less precision than controls. In addition, her performance in the find-and-replace task with repeated trials was clearly correlated with her starting position relative to the goal when entering the arena. P9's performance was worse when she entered the open-field arena through a door that was located on the same side of the arena as the goal, as compared to when she entered the arena through a door that was located on the opposite side of the arena. This result in the find-and-replace task seems more consistent with the use of a viewpoint-specific snapshot matching strategy than a viewpoint-independent (i.e., allocentric, spatial relational) representation of the goal location. Due to the position of the goal location 127 cm from the curtain on the left side of the arena and 163 cm from the open front of the arena, when she entered from the left (same) side, she could use either the curtain at the back of the arena (that was 237 cm away), the curtain on the right side of the arena (273 cm away) or the front of the arena (163 cm away) for a viewpoint-specific snapshot memory of the goal. In contrast, when entering from the right (opposite) side, the curtain on the left side was only 127 cm directly behind the goal location. Our data suggest that the closer the goal location was from the background scene used to define the “matching view,” the more accurate was her estimation of the goal location. The estimations of control participants did not exhibit this variation. As shown previously by imaging studies in humans, the parahippocampal cortex seems to contribute to the representation of visual scenes in an observer-centered (viewpoint-specific) rather than a world-centered (viewpoint-invariant) reference frame (Epstein et al., 2003; Epstein, 2008; Park and Chun, 2009). The greater involvement of the parahippocampal cortex in visually-guided scene-based navigation is also suggestive of its specificity to more rigid forms of scene representation (Zhang and Ekstrom, 2013). Accordingly, although some of our current findings are consistent with Bohbot's previous experimental results, we favor the interpretation that the parahippocampal cortex enables a relatively imprecise learning of a single location based on a viewpoint-specific memory representation. It can be argued that extraordinary claims require extraordinary evidence; we do not believe that the persistence of such limited spatial learning in patients with hippocampal damage constitutes sufficient evidence to invalidate the well-established theory that the mammalian hippocampus is necessary for the elaboration of an allocentric, viewpoint-independent, representation of space. Instead, the more parsimonious interpretation is that humans with hippocampal damage, like rodents with hippocampal lesions (Pearce et al., 1998), are able to use alternative spatial strategies to identify a single location in space.

#### Nine-box maze and lightboard tabletop experiments

Two studies investigated the effects of hippocampal damage on allocentric spatial memory using tabletop experimental designs and a testing procedure in which the subject had to move around the testing apparatus between stimulus presentation and choice. In a first study, Abrahams and colleagues demonstrated a deficit in allocentric spatial memory in patients with right hippocampal damage (Abrahams et al., 1997). In this task, nine identical containers were regularly arranged in a circle on a square board (78 × 78 cm), at a distance of 19 cm from each other. The experimenter selected four to-be-remembered objects and hid them in four to-be-remembered locations, while the subject was watching. The subject then changed position relative to the apparatus, by moving to one of the three other positions around the square experimental design between the presentation and memory phases. Patients with right hippocampal damage were impaired as compared to controls when asked to point to the containers that were hiding the objects. In contrast, they performed as well as controls when asked to select the images of the four hidden objects printed on a piece of paper. These findings are consistent with the view that the human hippocampus contributes to allocentric spatial learning and memory.

In a second study, Holdstock and colleagues used a 60.5 × 91 cm test board with 25 LEDs arranged in a random manner (Holdstock et al., 2000). They found that patient YR with bilateral hippocampal damage was more impaired at recalling the position of a single light at 20 and 60 s delays under conditions which strongly encouraged the use of an allocentric frame of reference than under conditions which forced the use of an egocentric frame of reference. It is interesting to note that in this task, subjects were not allowed to look at the test board while moving to the new viewing location, and that they were also required to perform an interference task by reading a passage of prose during the retention interval until approximately 3 s before the end of the delay. Surprisingly, YR was not impaired in the allocentric condition with a 5 s delay, suggesting that the deficit in allocentric spatial memory was delay-dependent. However, as YR's performance was not impaired as compared to controls in a mental rotation control task, it is also possible that YR's preserved mental rotation abilities enabled her to recall the location of a single light in absence of an effective interference procedure over the 5 s delay. As noted by the authors, it was also unclear whether a clear egocentric spatial memory deficit would be observed if the number of egocentric spatial memory associations to be remembered was increased. In our view, increasing the number of egocentric memories, and building relations between these memories as was suggested, would be the basis for the elaboration of an allocentric spatial representation. In sum, Holdstock et al.'s findings were also largely consistent with the view that hippocampal damage impairs memory for allocentric spatial information in humans.

#### Virtual reality experiments

Although the necessity of using virtual reality paradigms to perform functional brain imaging studies is undeniable, one must be cautious when drawing conclusions regarding the specific involvement of the hippocampus in particular cognitive processes based on virtual reality studies alone (Ravassard et al., 2013). Indeed, high activity can be observed in the medial temporal lobe (both in the hippocampus and the parahippocampal cortex) even during periods of rest, when subjects are not instructed to perform any particular task, and is presumably the result of mind wandering (Stark and Squire, 2001). Accordingly, if the activity of the hippocampus is relatively equal in all experimental conditions, or when the subject is at rest and free to think about whatever s/he wants, the absence of a differential activation between conditions cannot necessarily be interpreted as the absence of hippocampal involvement in the target condition. Nevertheless, and despite the inherent differences between real-world and virtual-reality paradigms, a number of studies of patients with hippocampal damage (Morris et al., 1996; Bohbot et al., 2004; Morris and Mayes, 2004; Parslow et al., 2004; Goodrich-Hunsaker and Hopkins, 2010; Goodrich-Hunsaker et al., 2010), as well as neuroimaging studies of healthy participants (Maguire et al., 1998; Bohbot et al., 2007; Suthana et al., 2009), generally support the view that the human hippocampus contributes to allocentric (viewpoint-independent) spatial memory processes. These neuroimaging findings are supported by electrophysiological recordings in epilepsy patients showing that cells responding to specific spatial locations are present primarily in the hippocampus, whereas cells that respond to specific views of landmarks are primarily found in the parahippocampal region (Ekstrom et al., 2003).

***In sum***, our current findings together with an abundance of experimental evidence from several lines of research in rodents, monkeys and humans are consistent with the view that the mammalian hippocampus plays a fundamental role in allocentric, spatial relational learning and memory processes, in particular in allowing precise metric coding, a hallmark of cognitive maps. In addition, as was previously shown in rodents and monkeys, some spatial learning remains possible following hippocampal dysfunction in humans. However, the spatial representations that can be elaborated by the rest of the brain appear more limited both in their precision (metric coding) and in the number of items that can be maintained in memory (capacity).

### Comparison with previous studies of short-term/working memory

In their first report on patient HM, Scoville and Milner focused on the finding that the hippocampus is essential for the formation of long-term memories of current experience (Scoville and Milner, 1957). However, in their description of patients with severe memory deficits, they also suggested that the hippocampus was necessary to maintain information over short delays (up to at least 15 min Milner et al., 1998), in the presence of distractive information. The effect of interference during the active maintenance of memory has often been overlooked when studying the hippocampus, and for many years working memory was essentially defined as the kind of memory that is spared in amnesia, and thus necessarily supported by structures outside the medial temporal lobe independently of the hippocampus. In contrast, the hippocampus was universally believed to be essential for the formation of long-term memory (Drachman and Arbit, 1966; Alvarez and Squire, 1994; Milner et al., 1998; Morris, 2007; Shrager et al., 2008). However, this popular view has been challenged (Ranganath and Blumenfeld, 2005; Yonelinas, 2013) and recent data suggest that the hippocampus is also involved in the active maintenance of information to support working memory processes (Olson et al., 2006a,b; Axmacher et al., 2007; Cowan, 2008; Cashdollar et al., 2009; Faraco et al., 2011; Race et al., 2013; Yonelinas, 2013; Yee et al., 2014).

Our current findings with P9 are in agreement with those studies demonstrating the role of the hippocampus in working memory processes. P9's performance was essentially flawless in the repeated-trials conditions with one, two or three color(s) to remember over 10 trials in the absence of interference. With our trial-unique procedure, in the absence of interference, P9 performed as well as controls with one color. For two or three items, however, her performance decreased significantly more than that of controls with interference. This finding is consistent with previous results showing that patients with medial temporal lobe damage are impaired at remembering visual information across short delays when memory load is increased (Jeneson et al., 2011, 2012). However, in presence of interference P9 was only able to perform four errorless trials out of 10 trials in the trial-unique procedure with one color to remember; she was unable to perform a single errorless trial with two or three colors. This pattern of results is clearly inconsistent with the view that visual working memory capacity is intact after damage to medial temporal lobe structures (Shrager et al., 2008; Jeneson et al., 2012). Instead, our data suggest that the medial temporal lobe contributes to the representation and active maintenance of sensory information, in parallel to the processing that might be taking place in sensory and other associational cortical areas. When the same color information had to be retained over repeated trials, active maintenance processes in extra-hippocampal cortical areas might be sufficient to support behavioral performance as the “new” information experienced on each encoding trial is the same as the “old” information already maintained. In contrast, when the specific information to be maintained must be updated on every trial, cortical representations might be effective, but extremely limited in capacity; P9's performance was equal to that of controls with one color, but significantly worse with two or three colors. When an interference procedure is further added during the retention interval, cortical areas might be unable to maintain an active representation of the information to be maintained while at the same time processing new information necessary for the performance of the interfering task. We suggest that a functional hippocampus might enable the parallel processing of information, and thus the maintenance of an active representation of the information to be remembered, while allowing subjects to simultaneously perform an interfering task. In sharp contrast with classical views (Baddeley, 2003), the hippocampus might actually be the central component of the brain's “working memory system.” This interpretation is consistent with Olton's theory that the hippocampus is involved in processing behaviors that require a short-term memory representation of flexible stimulus-response associations that are highly susceptible to interference, irrespective of the type of material (Olton et al., 1979).

## Conclusion

Our findings with a profoundly amnesic patient (P9) are consistent with the theory that the human hippocampus plays a key role in allocentric, spatial relational learning and memory. Although P9 was able, in some cases, to determine the general area where a single goal was located, the precision with which she was able to find one location was two to three times less good than that of controls. In addition, P9's cognitive deficits extended beyond spatial processing, as she was also impaired in trial-unique color learning, irrespective of memory load and interference conditions, although the impairment was greater in presence of interference. These findings are consistent with the theory that the hippocampus plays a key role in working memory requiring the maintenance of information particularly in presence of interference. Altogether, these findings are consistent with the theory that the hippocampus contributes to the integration or binding of multiple items, in order to produce high-resolution/high-capacity representations of spatial and non-spatial information in the service of short-term/working and long-term memory.

## Author contributions

Pamela A. Banta Lavenex and Pierre Lavenex were responsible for the conception and design of the work, acquisition, analysis and interpretation of the data, and drafting of the work. Françoise Colombo and Farfalla Ribordy Lambert were responsible for the acquisition of the data. Pamela A. Banta Lavenex, Françoise Colombo, Farfalla Ribordy Lambert and Pierre Lavenex have all critically revised the manuscript for intellectual content, approved the manuscript for publication, and agreed to be accountable for all aspects of the work.

### Conflict of interest statement

The authors declare that the research was conducted in the absence of any commercial or financial relationships that could be construed as a potential conflict of interest.
